# Diagnosis and treatment of mycotic aneurysms secondary to cardiac hydatid cyst: An unusual cause of multiple cerebral strokes in a 23-year-old female

**DOI:** 10.1016/j.radcr.2023.06.047

**Published:** 2023-07-07

**Authors:** Sara El Malih, Manar Ezzahi, Oughebbi Ismail, Amal Akammar, Nizar El Bouardi, Meriem Haloua, Badreeddine Alami, Meryem Boubbou, Mustapha Maaroufi, Moulay Youssef Alaoui Lamrani

**Affiliations:** a Department of Radiology and Interventional Imaging, CHU Hassan II Fez, Sidi Mohammed Ben Abdellah University, Centre Hospitalier Hrazem, BP:1835 Atlas, Fès, Avenue Hassan II, Fes 30050, Morocco; b Department of Radiology Mother and Child, CHU Hassan II Fez, Sidi Mohammed Ben Abdellah University, Fez, Morocco; c Department of Cardiovascular Surgery, Ghassani Hospital, Fez, Morocco

**Keywords:** Mycotic aneurysms, Ischemic stroke, Cerebral Hemorrhage

## Abstract

Mycotic aneurysms (MA) are a rare but severe complication of systemic infections, carrying a high risk of rupture, hemorrhage, sepsis, and potential multiple organ failure. Intracranial arteries are often affected and present a significant mortality risk due to cerebral bleeding and ischemic strokes. In this paper we describe the case of a 23-year-old female patient who presented with a sudden onset of right hemiparesis, followed by loss of consciousness. Cerebral imaging revealed multiple infarcts in both hemispheres and intracranial hemorrhage secondary to ruptured pseudoaneurysms. On transthoracic echocardiogram, she was found to have a left ventricular cardiac cyst with mobile vegetations potentially responsible for mycotic aneurysms and cerebral strokes. The patient underwent endovascular embolization for the mycotic aneurysms and cardiac surgery for the left ventricular cyst with satisfying clinical outcomes. Postsurgical analysis revealed the cyst to be of hydatid (Echinococcus) origin.

## Introduction

Mycotic aneurysms (MA) are irreversible arterial dilatations caused by an invading infectious organism, which weakens and destroys the artery wall leading to infectious arteritis, and carrying a high risk of rupture, hemorrhage, sepsis, and potential multiple organ failure [[1], [2], [3], [4]]. The name “mycotic” refers to the aneurysms' mushroom-like appearance, rather than their underlying microbiological etiology [5,6].

Intracranial mycotic aneurysms (ICMAs), are an uncommon inflammatory neurovascular disease that account for 0.7%-6.5% of all intracranial aneurysms [7]. They usually occur at terminal artery branches and have specific angiographic characteristics, natural history and clinical findings. Since spontaneous rupture causes subarachnoid and intracerebral hemorrhage, they are associated with considerable morbidity and mortality, with rates ranging from 60% to 90% in earlier case studies to 12%-32% in more recent literature reviews [8,9]. The risks are significantly increased in immune-compromised hosts, such as patients with HIV, diabetes, cancer or those undergoing chemotherapy [10].

Computed tomography angiography (CTA) remains the diagnostic and monitoring modality of choice

In this case report, we describe the diagnosis and management of intracranial MAs complicated by bilateral hemorrhagic and ischemic strokes, using CTA and interventional radiology.

## Case presentation

We report the case of a 23-year-old woman with no previous medical history of cardiovascular disease, who presented to the emergency department for sudden onset of right hemiplegia, followed by loss of consciousness.

On admission, the patient was unconscious with a Glasgow Coma Scale (GCS) of 9. Her remaining vital signs were stable, with a blood pressure of 120/70 mm Hg, heart rate of 70 bpm, and capillary blood glucose of 1.2 g/L. Neurological examination found right hemiplegia.

**Fig. 1:**
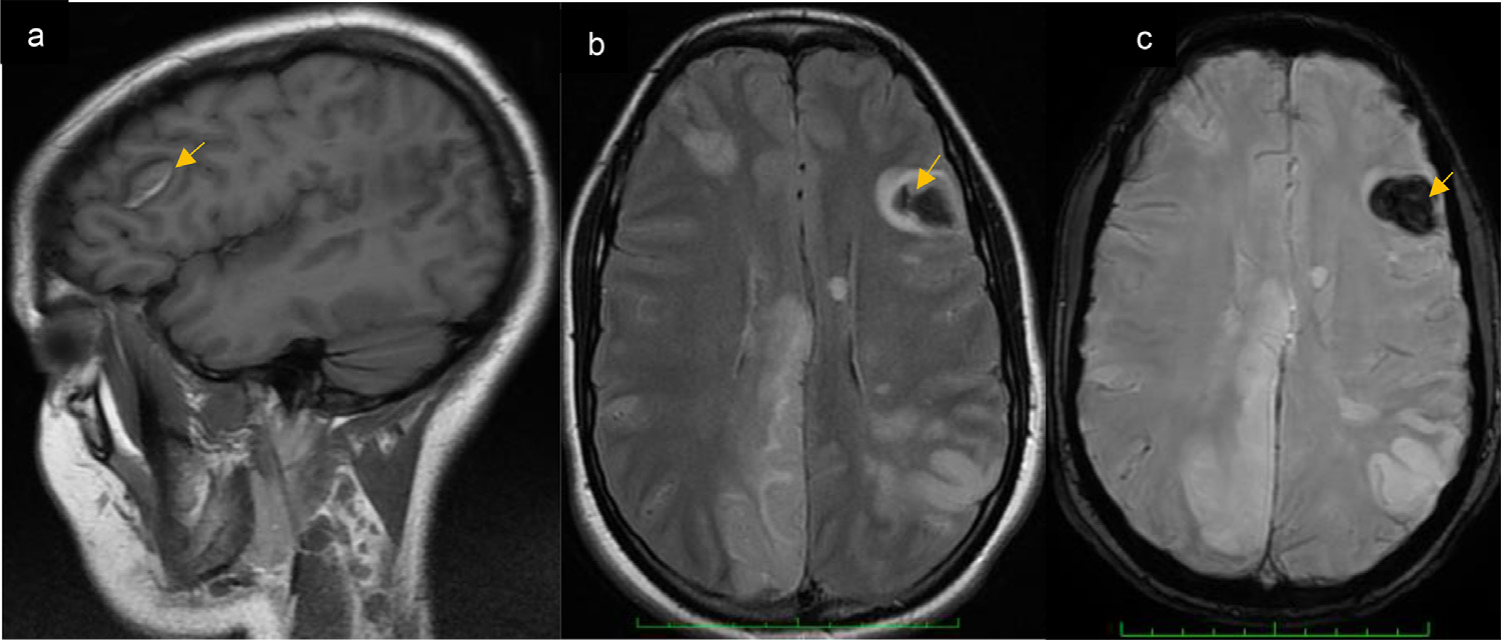
(A) Sagittal T1WI, (B) Axial FLAIR MR, and (C) T2*WI showing a left frontal lobe hemorrhage.

A standard nonenhanced computed tomography (NECT) was performed, and showed a left frontal lobe hemorrhage and bilateral hemispheric infarcts. (Fig. 1).

**Fig. 2:**
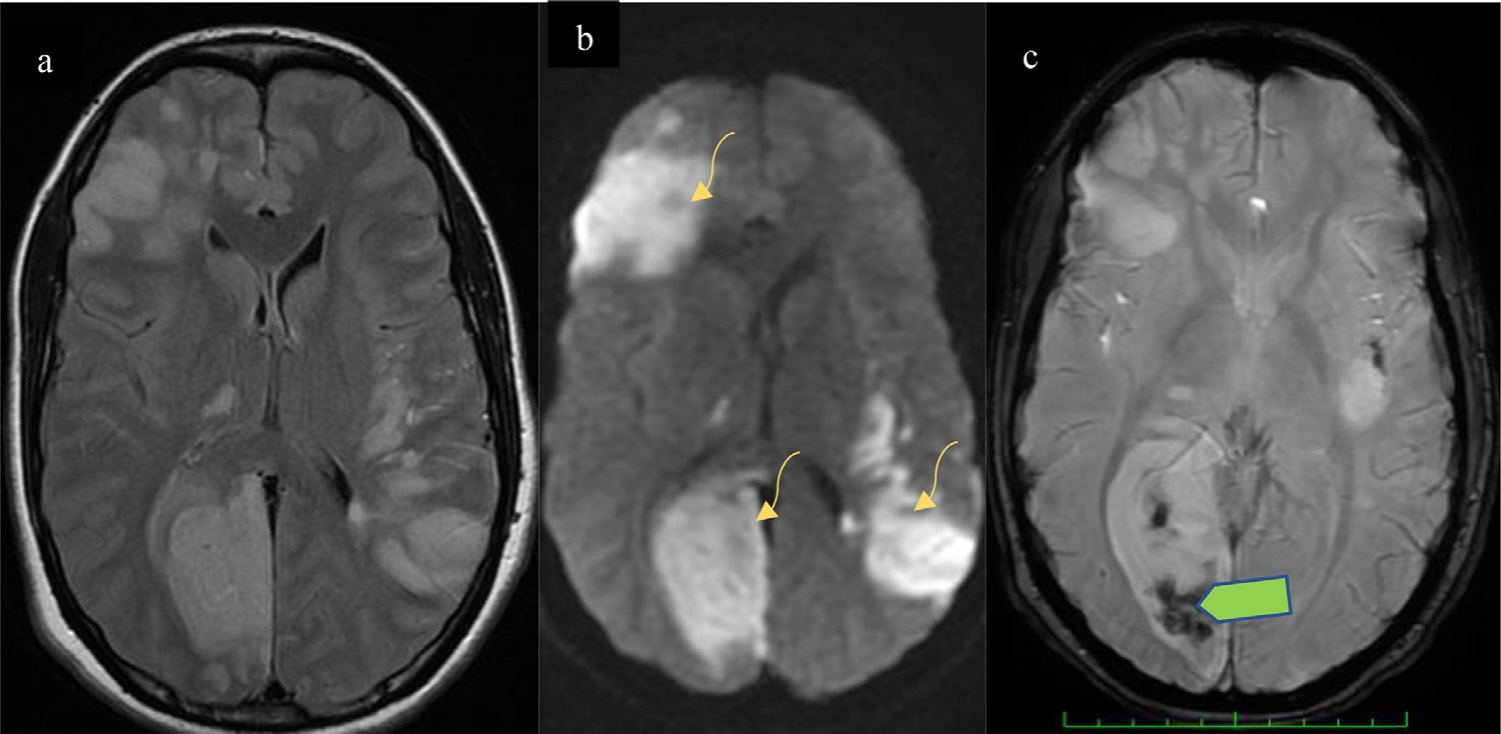
(A) Axial Flair MR showing multiple focal hyperintensities, with restricted diffusion in DWI sequence (B) corresponding to bilateral infarcts, T2*WI (C) demonstrates hemorrhagic transformation (Green arrow).

**Fig. 3:**
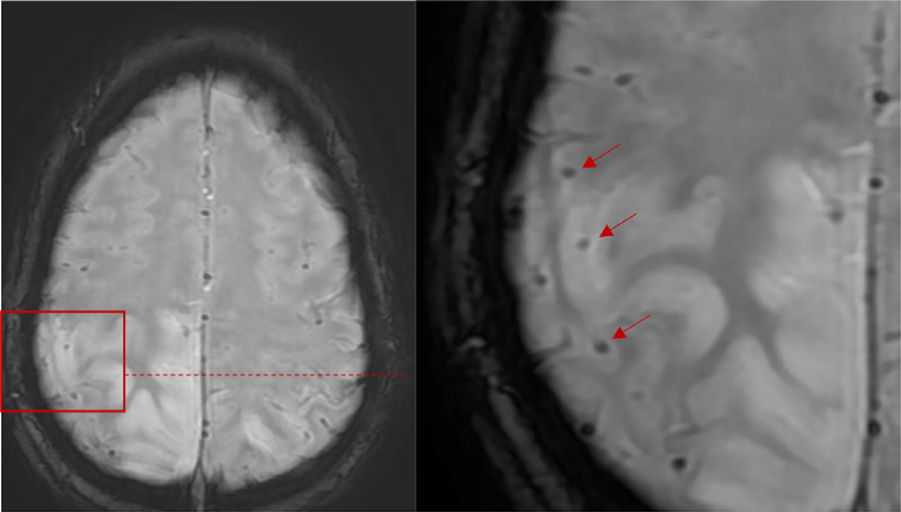
Axial T2* WI demonstrating multiple hypo-intense foci: “blooming black dots” corresponding to microbleeds.

**Fig. 4:**
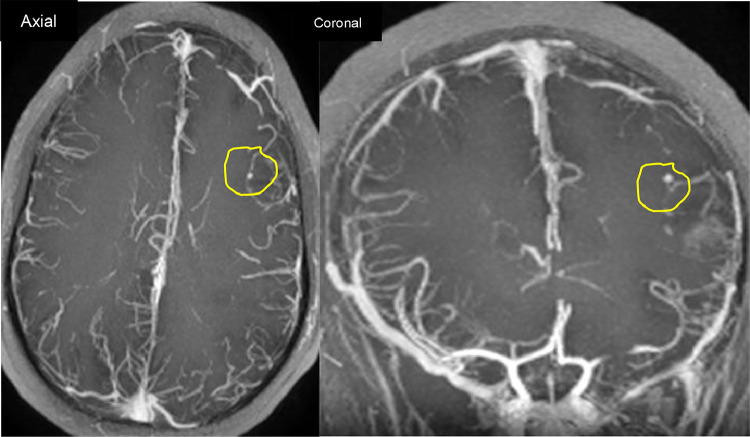
3DT1C+ MR showing a left frontal saccular pseudoaneurysm (Yellow circle).

MRI was performed the same day and revealed additional hemorrhagic transformation within the infarcted regions (Fig. 2) with diffuse cerebral microbleeds (Fig. 3). The T1weighted-image (T1W1) and postcontrast MRI sequences revealed a saccular pseudoaneurysm in the left frontal lobe (Fig. 4).

The patient was admitted to the intensive care unit (ICU) where electrocardiogram (ECG) and transthoracic echocardiogram (TTE) were performed as part of the standard cardiac workup for ischemic stroke. The ECG showed atrial fibrillation, and a large cystic lesion in the left ventricle was incidentally revealed on TTE. The left ventricular cystic lesion contained daughter vesicles, suggestive of a hydatid cyst. It also showed an irregular mobile septation on the surface indicative of possible vegetation.

Hematology demonstrated leukocytosis, and an elevated erythrocyte sedimentation rate (ESR). Blood cultures were negative for infectious organisms.

During hospitalization, the patient had a repeat episode of acute left hemiplegia coupled with acute neurological deterioration to a GCS of 6. A NECT brain scan and CTA of the circle of Willis and supra-aortic trunks was immediately performed.

**Fig. 5:**
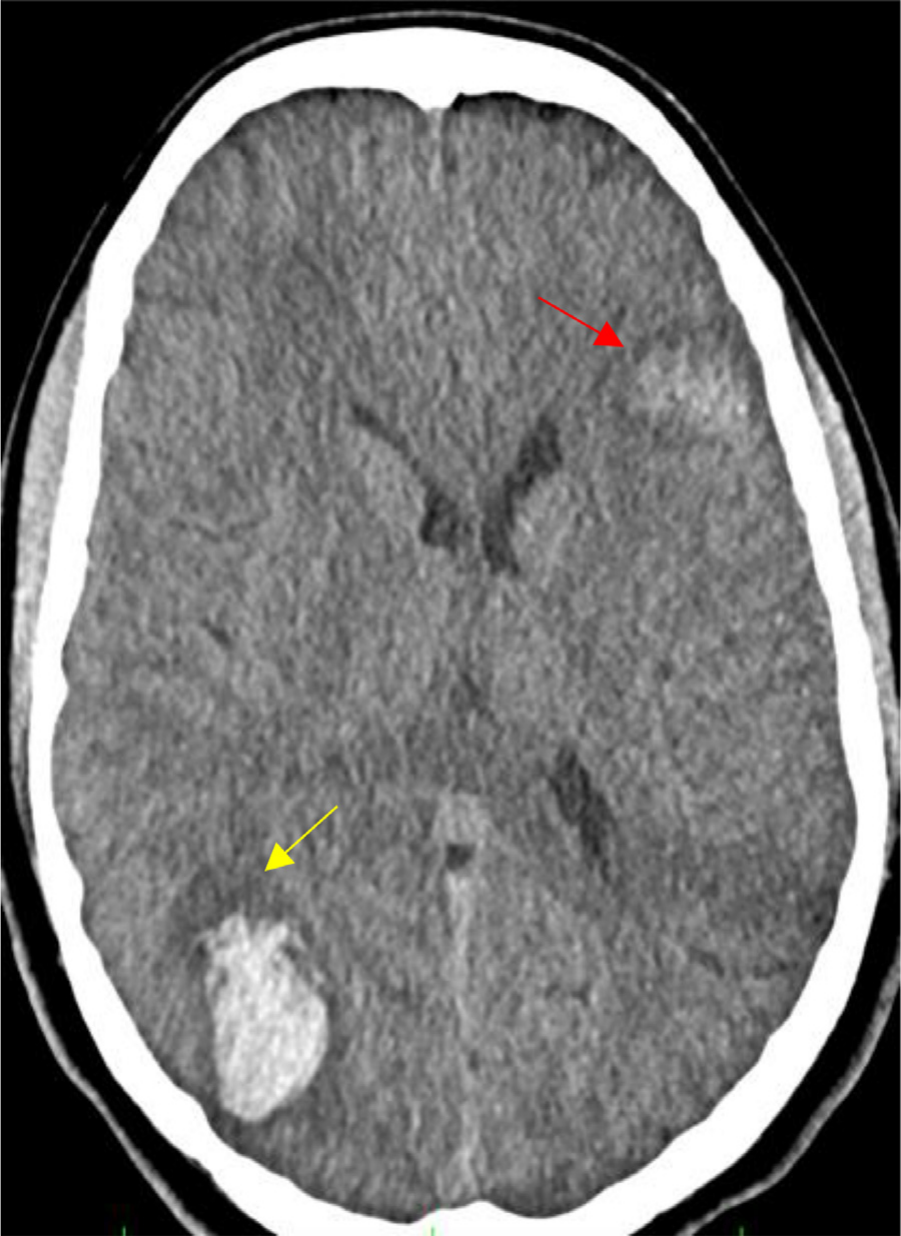
Axial NECT showing the appearance of a right parieto-occipital hematoma (yellow arrow). Note the regression of left frontal lobe hemorrhage (Red Yellow).

**Fig. 6:**
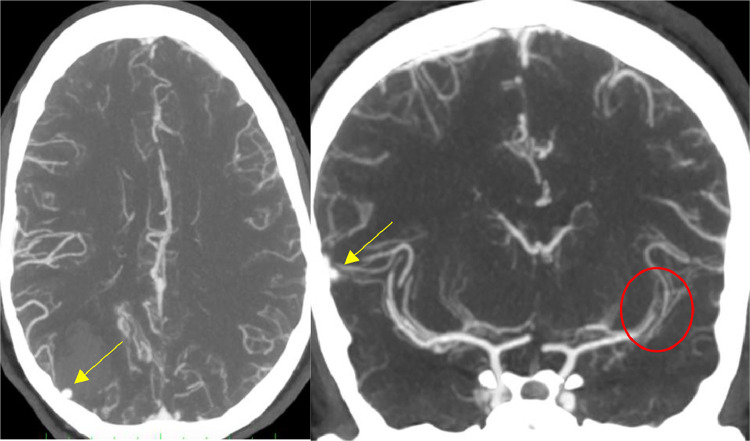
CTA of the brain, in axial (left) and coronal reconstruction (right), showing a saccular pseudoaneurysm in the right parieto-occipital lobe (Yellow arrow), and occlusion of the M3 segment of the left middle cerebral artery (Red circle).

The NECT scan showed a new occurrence of right parieto-occipital hemorrhage (Fig. 5) while the cerebral CTA revealed a new saccular cortical pseudoaneurysm adjacent to the lobar parieto-occipital hemorrhage with occlusion of the M3 segment of the left middle cerebral artery (Fig. 6).

**Fig. 7:**
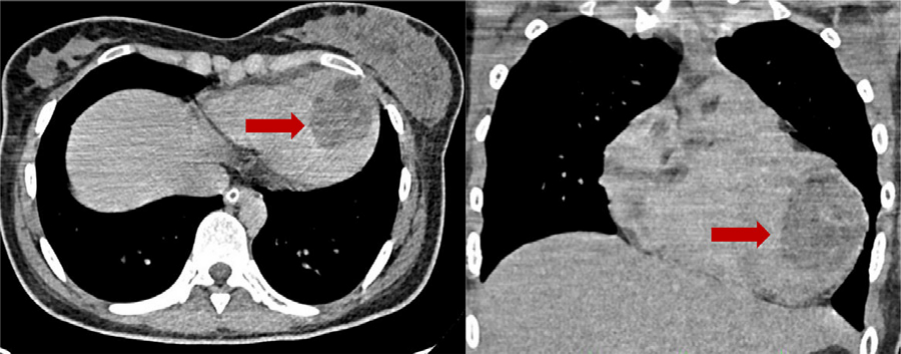
Thoracic CT scan, in axial and coronal reconstructions, showing well capsulated hypodense lesion seen in the myocardium of the left ventricle, containing multiple daughter cysts, suggestive of a hydatid cyst.

A thoracic CT scan showed a multivesicular cystic lesion in the left ventricle suspected of being a hydatid cyst (Fig. 7).

**Fig. 8:**
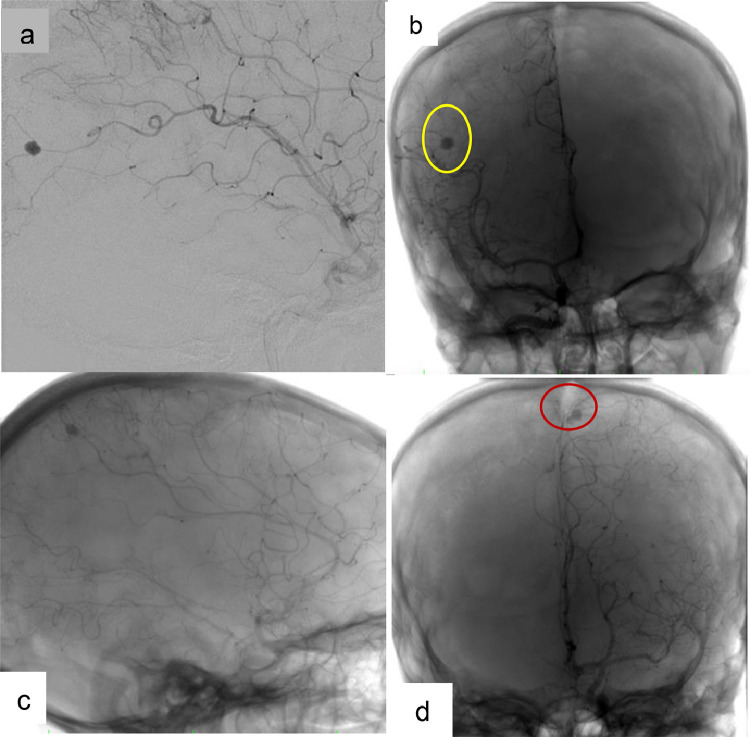
Cerebral DSA showing 2 pseudoaneurysms, in left parieto-occipital (A, B), and right frontal lobes (C, D).

**Fig. 9:**
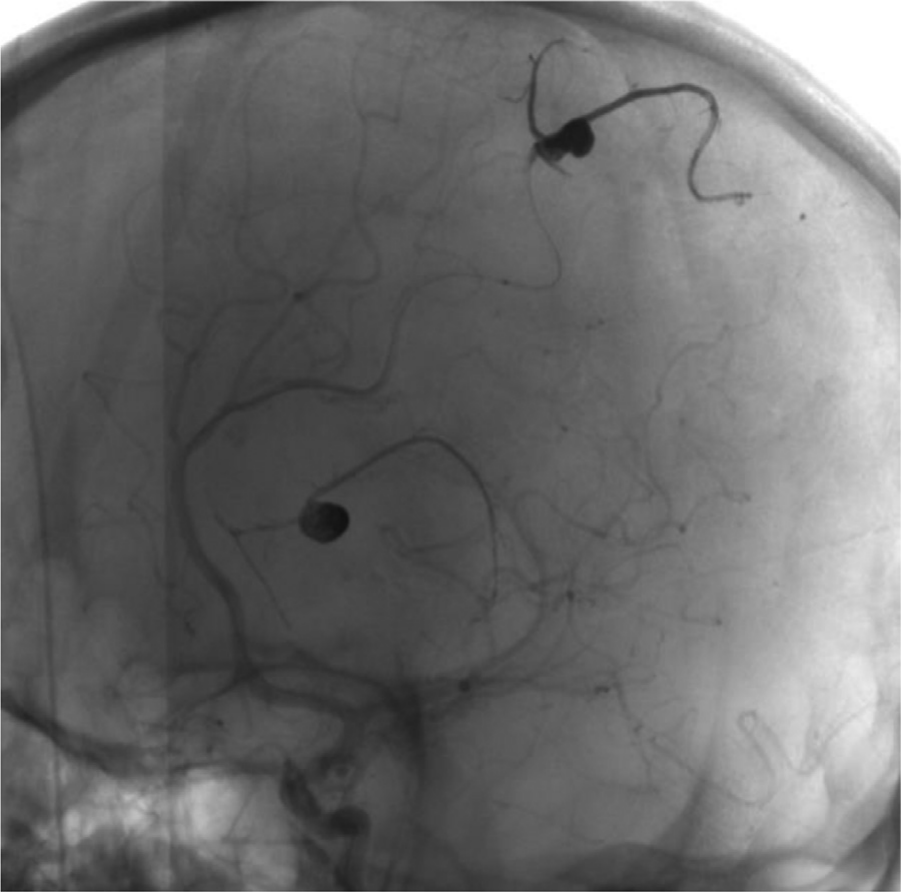
Cerebral DSA showing 2 pseudoaneurysms in the left parietooccipital lobe and the right frontal lobe.

**Fig. 10:**
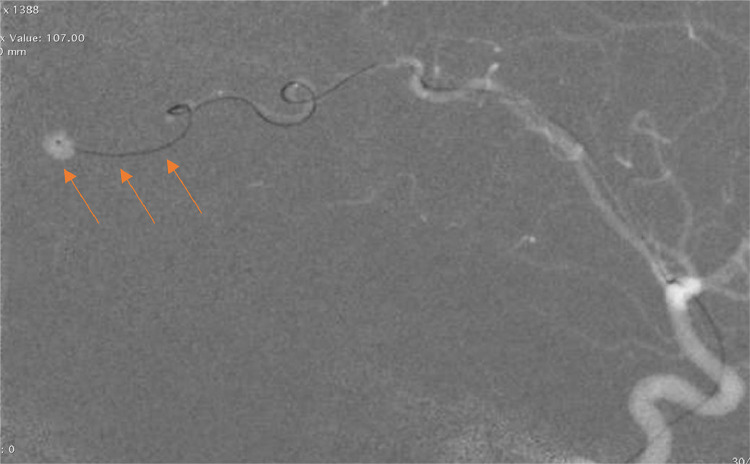
Cerebral DSA showing the coil embolization of the pseudoaneurysm.

Digital Subtraction Angiography (DSA) was performed the next day, for both diagnostic and therapeutic purposes, and revealed intracranial saccular pseudoaneurysms in right parietal and left frontal lobes. Embolization was performed the same day with satisfying clinical outcomes and a rapid improvement in the patient's state of consciousness (Fig. 8, Fig. 9, Fig. 10).

**Fig. 11:**
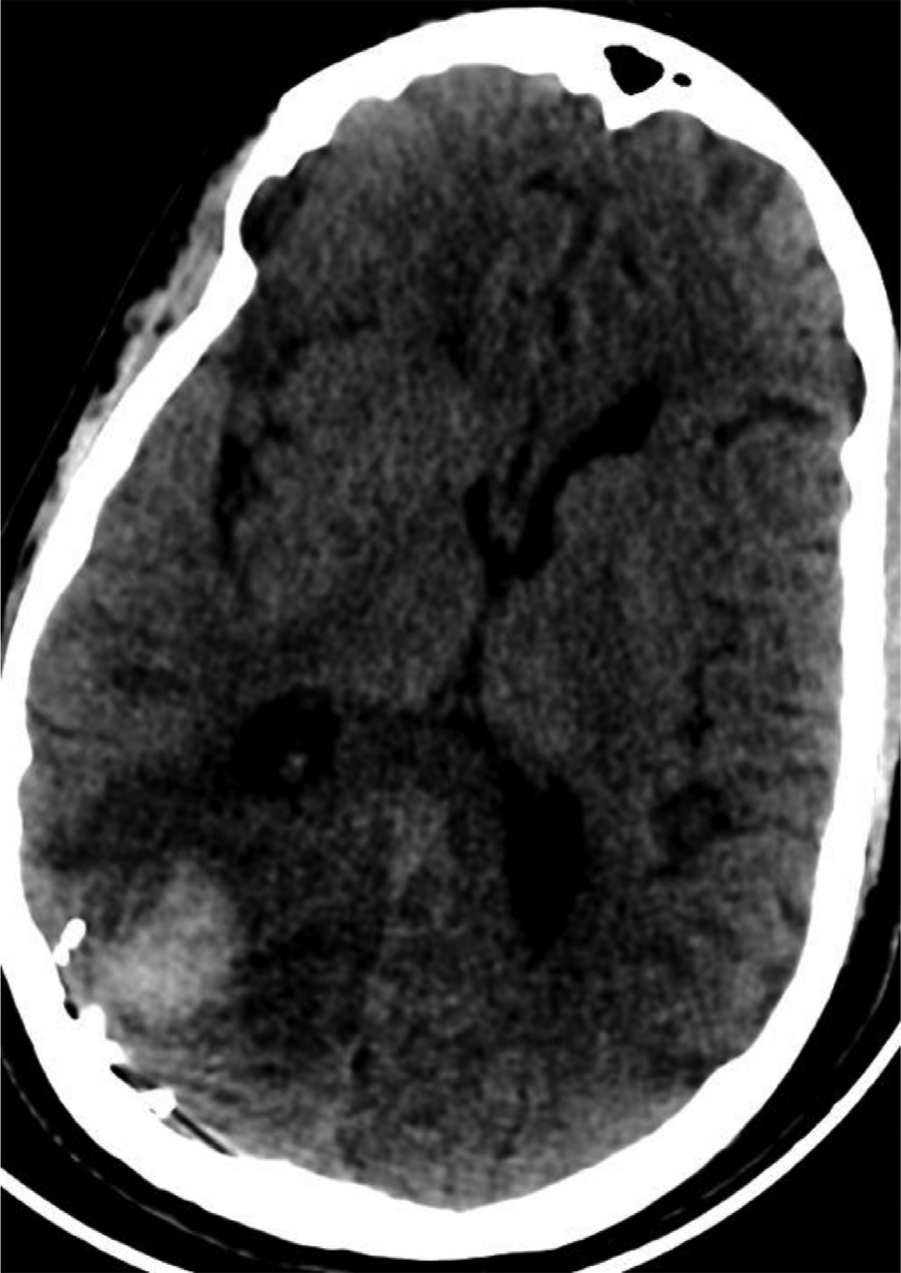
Follow-up NECT of the brain after coil embolization of the pseudoaneurysm.

Following embolization, a Cerebral NECT was performed with satisfying radiological results (Fig. 11).

On day “10” of hospitalization, the patient developed acute left lower limb ischemia due to embolic occlusion of the left popliteal artery. A percutaneous Fogarty thrombectomy was successfully performed with complete revascularization of the popliteal segment.

**Fig. 12:**
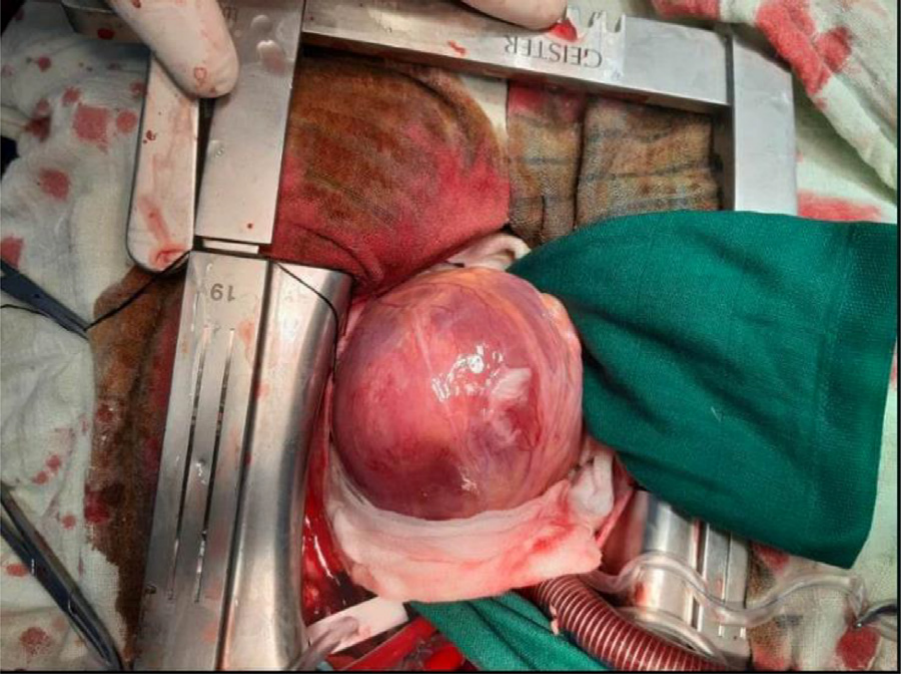
Perioperative image of the intracavitary cardiac cyst during the surgical procedure.

One month after initial hospitalization, the patient underwent surgical removal of the cardiac hydatid cyst to avoid reoccurrence of mycotic aneurysms and septic emboli (Fig. 12).

## Discussion

Mycotic aneurysms can be defined as aneurysmal dilations of the arterial wall, due to an underlying infectious etiology [6]. All arterial territories can be affected, however location in the cerebral arteries carries a near-fatal prognosis due to the complications of vascular occlusion and cerebral infarction and intra cranial hemorrhage due to their high risk of rupture [11].

Different mechanisms have been postulated for the development of MA, however the vasa vasorum theory is the pathogenic process most frequently observed. It proposes that bacteria from septic emboli escapes through the vasa vasorum and generates significant media and adventitial inflammation and necrosis. Infection then spreads throughout the body and leads to aneurysmal development [12], [13], [14].

Although a variety of bacteria, mycobacteria, viruses, and fungi can cause mycotic aneurysms, the principal organisms responsible are the Viridans group Streptococci (VGS) and Staphylococcus aureus by colonizing the cardiac valve endocardium.

In our case, the presence of an infected cardiac hydatid cyst was a predisposing factor for the patient's mycotic aneurysms. Cardiac involvement in hydatid disease is very rare, constituting only 0.5%-2% of all cases of hydatidosis even in endemic areas [15]. It can occur in any site within the heart, but the left ventricle is the most commonly involved, with high risk of rupture resulting in anaphylactic shock, tamponade, systemic or pulmonary embolization, and coronary branches constriction.

MAs present a variety of clinical manifestations, the most common symptoms are fever, and local aches relevant to localized vascular inflammation (back pain for aortic localization, and headaches for intracranial vessels) [16]. Because these symptoms are non-specific, many patients are classified as having a fever of unknown origin and are either mis- or under-diagnosed until serious complications such as sepsis, thrombosis, hemorrhage, or vascular rupture lead to emergent presentation.

For the diagnosis of MAs, a scoring system based on the presence of particular clinical and radiographic features has been proposed [17]. There are points awarded for the presence of clinical markers such as infective endocarditis, meningitis, orbital cellulitis, persistent fever, age under 45years, history of recent lumbar puncture, or radiographic evidence of aneurysm multiplicity; distal location, fusiform shape, irregular contours and size change [17].

CTA, magnetic resonance angiography (MRA), and digital subtraction angiography (DSA) are all options for cerebral vascular imaging to diagnose ICMA. With the introduction of Multidetector CT imaging (MDCT), the resolution of CTA has grown, allowing full visibility of the cerebral vascular tree with reduced contrast load and reduced risk of persistent neurologic impairments compared to DSA. MRA is another advanced imaging technology used to diagnose cerebral aneurysms, however, for aneurysms smaller than 3 mm, its sensitivity drops to 38% compared to CTA [18].

Indirect signs of ICMA, visualized on CT or cerebral MRI, include cerebral and/or subarachnoid hemorrhage, possibly associated with ischemic lesions, cerebral edema or hydrocephalus. These signs help to indirectly localize the topography of the aneurysm.

Throughout the last decade, the treatment of infective cerebral aneurysms has been split into 3 categories: medical, endovascular, and surgical [19]. This splitting has improved overall prognosis.

In our case, the patient received timely diagnosis and treatment for her acute stroke and ICMAs in hospital, which considerably improved prognosis.

## Summary

MA are uncommon but are almost always lethal if not treated properly. In our case the patient's development of left ventricular infective endocarditis due to Echinococcus infection caused disseminated disease resulting in multiple localized MAs which caused localized neurological impairments.

As a result, it is critical to investigate the existence of MAs, and CTA should be performed to rule them out. Medical therapy for MAs should be limited to those patients with unruptured aneurysms and should match the etiology. Patients should be continuously monitored with repeat cerebral imaging and endovascular intervention should be performed only once the patient is stable. Surgery is indicated as soon as there are signs of increased intracranial pressure.

## Ethics approval

Not applicable. All authors agreed for publication.

## Availability of data and materials

The data sets are generated on the data system of the CHU Hassan II of Fes, including the biological data and the interventional report.

## Patient consent

Written informed consent was obtained from the patient. A copy of the written consent is available for review by the Editor-in-Chief of this journal.
